# Current Methods for Skeletal Muscle Tissue Repair and Regeneration

**DOI:** 10.1155/2018/1984879

**Published:** 2018-04-16

**Authors:** Juan Liu, Dominik Saul, Kai Oliver Böker, Jennifer Ernst, Wolfgang Lehman, Arndt F. Schilling

**Affiliations:** ^1^Clinic for Trauma Surgery, Orthopedics and Plastic Surgery, University Medical Center Göttingen, Göttingen, Germany; ^2^Clinic for Plastic Surgery, Technische Universität München, München, Germany; ^3^Wuhan Union Hospital, Tongji Medical College, Huazhong University of Science and Technology, Wuhan, China

## Abstract

Skeletal muscle has the capacity of regeneration after injury. However, for large volumes of muscle loss, this regeneration needs interventional support. Consequently, muscle injury provides an ongoing reconstructive and regenerative challenge in clinical work. To promote muscle repair and regeneration, different strategies have been developed within the last century and especially during the last few decades, including surgical techniques, physical therapy, biomaterials, and muscular tissue engineering as well as cell therapy. Still, there is a great need to develop new methods and materials, which promote skeletal muscle repair and functional regeneration. In this review, we give a comprehensive overview over the epidemiology of muscle tissue loss, highlight current strategies in clinical treatment, and discuss novel methods for muscle regeneration and challenges for their future clinical translation.

## 1. Introduction

Skeletal muscle is one of the most abundant tissues in the human body. It accounts for 40%–45% of the total body mass and is necessary for generating forces for movement [1]. Up to a certain threshold, skeletal muscle has the capability of regenerating lost tissue upon injury [2]. Beyond this threshold, the remaining muscle tissue is unable to fully regenerate its function. This loss of skeletal muscle with lasting functional impairment is defined as “volumetric muscle loss” (VML) [3–5]. It can substantially impact the quality of life of patients by significantly reducing the functionality of the locomotion system [4].

Frequent reasons for skeletal muscle injuries are high-energy traffic accidents, blast trauma, combat injuries, surgical and orthopedic situations (e.g., after compartment syndrome or tumor resection), or contusion injury during sports that lead to an acute muscle tissue loss [6, 7]. Approximately 35–55% of sport injuries involve muscle damage at the myofiber level [8]. Those injuries that involve 20% or more of muscle loss of the respective muscle mass need reconstructive surgical procedures [9]. Progressive muscle loss can result from metabolic disorders or inherited genetic diseases such as Duchenne muscular dystrophy, Amyotrophic Lateral Sclerosis, and pediatric Charcot-Marie-Tooth disease [10–13]. Muscle atrophy can also be a consequence of peripheral nerve injuries, chronic kidney disease, diabetes, and heart failure [14, 15]. Up to 20% loss of muscle mass can be compensated by the high adaptability and regenerative potential of skeletal muscle. Beyond this threshold functional impairment is inevitable and can lead to severe disability as well as cosmetic deformities, which is why therapeutic options are in urgent demand for these patients [4, 5, 16, 17].

Muscle regeneration relies on a heterogeneous population of satellite cells, interstitial cells, and blood vessels and is mainly controlled through ECM proteins and secreted factors [18, 19]. Normally muscle mass is maintained by a balance between protein synthesis and degradation [20]. In most cases of VML, the regeneration capability of skeletal muscles is impeded, because necessary regenerative elements, mainly satellite cells, perivascular stem cells, and the basal lamina, are physically removed [21, 22]. Through denervation, protein degradation pathways (the proteasomal and the autophagic-lysosomal pathways) are activated. Therefore protein degradation rates exceed protein synthesis, which contributes to the muscle atrophy accompanied by gradual decrease of muscle wet weight and muscle fiber diameters [23, 24].

Revascularization is typically impaired. The following ischemic conditions favor fibroblast proliferation, fibrosis, and fibrotic scar tissue formation, which leads to further degeneration of the muscle [25]. The ECM composition and extent in scar tissues affect many aspects of myogenesis, muscle function, and reinnervation [26]. It can severely constrain motion and thereby aggravate the consequences of muscle tissue loss. Also in chronic muscle loss like Duchenne muscular dystrophy, fibrosis is a major problem [27]. Here, the consistent breakdown of myofibers cannot be fully compensated by satellite cell proliferation. The following inflammatory processes lead to an altered production of extracellular matrix (ECM) and consequent development of fibrosis and scar tissue formation [27–29]. This scar formation can be reduced either by injection of, for example, 5-fluorouracil and bleomycin, which antagonizes fibroblast proliferation and neoangiogenesis or by laser therapy with release of contracture and functional improvements after 6–12 months' treatment [30, 31]. Regeneration with regression of scar tissue and functional recovery can furthermore be optimized with fat grafting [32]. However, reducing scar formation is not enough for promoting muscle tissue repair and regeneration. This reinvigorates clinical and research efforts directed at replacing or regenerating larger volumes of muscle tissue.

## 2. Current Methods for Treating Muscle Tissue Loss in the Clinic

Current standard of care for VML is typically based on surgical intervention with autologous muscle graft and physical therapy. Further clinically used strategies include acupuncture and application of scaffolds.

### 2.1. Surgical Techniques

Surgical treatment for VML includes mainly scar tissue debridement and/or muscle transposition [33]. Autologous muscle transfer is commonly performed in a clinical situation, when there are large areas of muscle loss following trauma, tumor resection, or nerve injury, which impairs the irreplaceable motor function [34, 35]. The surgeons graft healthy muscle from a donor site unaffected by the injury to restore the lost or impaired function [36]. When no adjacent muscle is available because of high-level nerve injuries or severe trauma, autologous muscle transplantation together with neurorrhaphy, in the form of free functional muscle transfer, can be applied [37, 38]. The most popular autologous muscles are latissimus dorsi muscle and gracilis muscle. Latissimus dorsi muscle transfer has been shown to be safe and efficient for restoration of elbow flexion after injuries [34]. In the case of a synovial sarcoma affecting the right gluteus medius and minimus muscles, the function of the affected hip abduction could be fully reconstructed with a free neurovascular latissimus dorsi muscle transplantation [39]. Free gracilis muscle transfer is commonly utilized to restore elbow flexion after pan-brachial plexus injury [40]. It is also applied for muscle weakness after facial palsy or pelvic floor reconstruction [41, 42]. Although functional muscle flaps can lead to at least decent functional results, they cause substantial donor site morbidity and inadequate innervation [43]. Moreover, as many as 10% of these reconstructive surgeries result in complete graft failure due to complications such as infection and necrosis [44]. Sometimes, the source of autologous muscles for grafting is a problem, if the patient is severely injured.

### 2.2. Physical Therapy

Exercise has the ability to prevent a decrease of skeletal muscle mass [45]. Thus, in addition to surgical techniques, physical therapy is a noninvasive/minimally invasive way to promote muscle tissue repair and regeneration. It is especially used for rehabilitation after injuries and muscle tissue transfer, or to treat chronic muscle loss.

Physical rehabilitation aims at strengthening the remaining muscles. This has been shown to accelerate muscle healing/regeneration by modulating the immune response, release of growth factors, promoting vascularization, and reducing scar formation [46–48]. Functional performance of nonrepaired VML injured muscle could be significantly improved with physical rehabilitation in the form of voluntary wheel running [49]. Interventions to enhance angiogenesis including exercise and massage are potential strategies to accelerate new muscle formation in clinically transplanted muscle grafts or other surgical situations [50]. It has been reported that physical exercise can upregulate the IGF-1 signaling pathway and decrease myostatin in muscle tissue of animals and humans, thus preventing muscle atrophy [51–53].

Physical therapy can indeed improve muscle repair and recovery; however, it is unable to facilitate substantial muscle regeneration within the defect areas in VML. In addition, patients with severe diseases or injuries are frequently unable to make consistent exercise, which limits physical therapy as a treatment for VML.

### 2.3. Acupuncture

Acupuncture is a branch of traditional Chinese medicine, which has been widely used to treat various diseases around the world [54–56]. Electrical acupuncture treatment has been shown to suppress myostatin expression, leading to satellite cell proliferation and skeletal muscle repair [57]. Acupuncture plus low-frequency electrical stimulation (Acu-LFES) could enhance muscle regeneration and prevent muscle loss by replicating the benefits of exercise through stimulation of muscle contraction [58]. It is suitable for some patients with severe diseases, which are unable to perform exercise frequently. Acu-LFES was shown to counteract diabetes-induced skeletal muscle atrophy by increasing IGF-1 and thereby stimulating muscle regeneration [58]. Application of Acu-LFES for the treatment of diabetic myopathy and muscle loss induced by chronic kidney disease showed good functional improvement of the muscle [58, 59]. The underlying mechanism includes activation of M2 microphages and reversing mRNA expression levels of the E3 ubiquitin ligase atrogin-1.

Similar to physical exercise, acupuncture improves muscle function restoration and stimulates muscle regeneration especially in patients with muscle atrophy after chronic diseases. However, there is limited success for the regeneration of large volume muscle defects after trauma or tumor resection. Furthermore, more work needs to be done to determine the optimal timing and intensity of Acu-LFES as a standard treatment for muscle atrophy.

### 2.4. Biological Scaffolds

*Biological scaffolds* composed of extracellular matrix (ECM) proteins are commonly used in regenerative medicine and in surgical procedures for tissue reconstruction and regeneration. The scaffolds can promote the repair of VML by providing a structural and biochemical framework [60]. For smaller amounts of muscle loss, several tissue-derived scaffolds have been tested in animal models and translated into the clinic for surgical application [6]. Xenogeneic extracellular matrix and autologous tissue have been utilized to restore functional muscle and simultaneously generate a biological niche for recovery [61]. A multilayered scaffold made of ECM derived from porcine intestinal submucosa has been applied for reconstruction of vastus medialis muscle in patients [16]. The patient showed marked gains in isokinetic performance 4 months after surgery and new muscle tissue at the implant site was demonstrated by computer tomography. Porcine small intestinal submucosa-extracellular matrix has also been utilized for the treatment of abdominal musculoskeletal wall defects, where it was sutured at the defect corners and subcuticularly closed with a vicryl-suture [61]. Also, porcine ECM from urinary bladder has been implanted in an attempt to treat VML in human beings [60]. Functional improvement with formation of muscle tissue was observed in three of the five human patients in this study.

However, allograft or xenogeneic scaffolds can still induce adverse immune response after decellularization and there might be potential risk of infectious disease transmission. Therefore, there is a clinical need to develop new strategies that can facilitate safe bigger muscle tissue repair and regeneration.

## 3. Developing Technologies for Muscle Tissue Engineering and Regeneration

To address remaining clinical problems and explore novel strategies for muscle tissue engineering and regeneration, new technologies have been investigated intensively. While tissue bioengineering approaches aim to construct complex muscle structures in vitro for subsequent implantation and replacement of the missing muscles, tissue regeneration approaches develop tissue-like scaffolds that can be implanted to enhance new muscle formation from remaining tissue in vivo [62]. Both approaches mainly rely on combinations of scaffolds, cells, and molecular signaling with differing focus.

### 3.1. Scaffold-Based Strategies

Biomaterials can provide chemical and physical cues to transplanted cells or host muscle cells to enhance their survival, promote their functional maturation, protect them from the foreign body responses, and recruit host cells and regenerate muscle tissues [63]. Biological scaffolds are used in a variety of clinical tissue engineering applications and have been studied in preclinical skeletal muscle VML injury models frequently over the last decade. They are mainly made of natural polymers, synthetic polymers, or ECM and attempt to create a microenvironment niche to favorably control the behavior of resident cells.

*Natural polymers* such as alginate, collagen, and fibrin have been utilized extensively in skeletal muscle engineering [64–66]. They possess intrinsic bioactive signaling cues to enhance cell behavior [67–69]. Alginate gels with a stiffness of 13–45 kPa were found to maximize myoblast proliferation and differentiation [70]. Freeze-dried collagen scaffolds facilitated the integration of aligned myotubes into a large muscle defect, which were capable of producing force upon electrical stimulation [71]. Collagen could also supply necessary growth factors to the wound site to increase muscle cell migration [72, 73]. Fibrin gels were reported to promote myoblast survival and differentiation into myofibers when integrated in tissues [74]. Fibrin scaffolds with microthread architecture were also shown to support the healing of VML in mouse models [75].

As the natural polymer only offers limited mechanical stiffness and can be easily degraded, a variety of* synthetic materials* have been used for skeletal muscle regeneration such as PGA, PLA, and PLGA [66, 76–78]. Myoblasts seeded onto electrospun meshes with aligned nanofiber orientation can fuse into highly aligned myotubes [78]. Furthermore, synthetic scaffolds can be easily engineered to facilitate the controlled release of growth factors for inducing muscle regeneration [75, 79]. The main disadvantages include typically poorer cell affinity compared to natural polymers and the risk of stimulation of a foreign body response by the polymer or its degradation products [79].

To improve regeneration of muscle tissues, the in vivo microenvironment of the scaffolds ideally would mimic native tissues and thereby facilitate remodeling of the neotissue [80]. An attractive approach for the repair of VML is therefore the transplantation of a myoinductive decellularized scaffold that attracts the cells required for myogenesis from the host. That is why muscle-derived* ECM scaffolds* are popularly investigated. These ECM scaffolds can fill the defect and restore morphology temporarily [17]. They can further be filled by bone-marrow derived mesenchymal stem cells (MSCs) after implantation. This enriched matrix gains more blood vessels and regenerates more myofibers than “conventional” extracellular matrix [17, 81]. Indeed, hydrogels derived from decellularized skeletal muscle matrix have been shown to enhance the proliferation of skeletal myoblasts when injected into an ischemic rat limb [82]. An alternative method could be to utilize minced skeletal muscle tissue that has not been decellularized, which has been reported to show better muscle regeneration than devitalized scaffolds [83]. Comparable to muscle-derived matrix, small intestinal submucosa-extracellular matrix can lead to contractile sheets of skeletal muscle with comparable contractile force [61]. For in vitro muscle tissue engineering, rat myoblasts have also been preconditioned on a porcine bladder acellular matrix in a bioreactor and then implanted in nude mice at a muscle defect to restore muscular tissue [80].

Another obstacle in muscle regeneration is the musculotendinous junction. This can be partly restored in absence of implanted cells by extracellular matrix-based platforms that have been shown to withstand half of the force of the contralateral site after complete resection in a mammalian model [80]. The newly formed muscle cells have shown better adherence to 3D polyurethane-based porous scaffolds with low stiffness and larger roughness values [84].

### 3.2. Cell-Based Strategies

Muscle fiber regeneration is performed by cells and consequently cell-based strategies for regeneration have been pursued [83, 85]. The cell types utilized for treating muscle loss mainly include myoblasts, satellite cells (SCs), mesoangioblasts, pericytes, and mesenchymal stem cells (MSCs) [86–88]. The most well characterized muscle stem cell is the satellite cell (SC). SCs are able to contribute extensively to the formation of new muscle fibers [86, 89]. SCs transplanted into dystrophin-deficient mdx mice yielded highly efficient regeneration of dystrophic muscle and improved muscle contractile function [90]. Unfortunately, in vitro expansion of SCs results in significant reduction of their ability to produce myofibers in vivo [91] and consequently, obtaining a sufficiently large number of fresh SCs for clinical application is impractical [92]. Myoblasts have been used for reconstructing muscle tissue defects with a variety of scaffolds [87, 93, 94]. They were shown to functionally integrate into the existing musculature of the host. Injection of a larger number of myoblasts into muscles showed promising results for the treatment of dystrophin-deficient models [95]. Also MSCs could be involved in myotube formation through heterotypic cell fusion after myogenic gene activation [88]. Mesoangioblasts and pericytes have been studied for treating muscular dystrophy, which resulted in increasing the force [96]. They have also been utilized in tissue engineered hydrogel carriers, with some reported success for promoting muscle regeneration [97].

Stem-cell-based therapies provide notable therapeutic benefits on reversing muscle atrophy and promoting muscle regeneration. Stem cell therapy (e.g., umbilical cord blood stem cell transplantation) showed positive results for treating Duchenne muscular dystrophy [98]. After application of stem cells, an increase of dystrophin positive muscular fibers was found. Biopsies of calf muscle showed growing myoblasts cells and muscular tubes and an improvement in arms and legs during physical examination was reported.

### 3.3. Molecular Signaling Based Strategies

Beside cues from the ECM, also a diversity of stimulatory and inhibitory growth factors such as IGF-1 and TGF-ß1 can drive endogenous skeletal muscle regeneration by activating and/or recruiting host stem cells [22]. They can be loaded on scaffolds for controlled delivery to the injured areas [72, 99]. Sustained delivery of VEGF, IGF-1, or SDF-1a was shown to enhance myogenesis and promote angiogenesis and muscle formation [73, 100–102]. Rapid release of hepatocyte growth factor (HGF) loaded on fibrin microthread scaffolds promoted remodeling of functional muscle tissue and enhanced the regeneration of skeletal muscle in mouse models [75]. Combination therapy of h-ADSCs and bFGF hydrogels resulted in functional recovery, revascularization, and reinnervation in lacerated muscles with minimal fibrosis [103]. Furthermore, PEDF peptide was reported to promote the regeneration of skeletal muscles [104].

Research into the pathogenesis of sarcopenia as one of the most frequent muscular diseases has elucidated different molecular pathways. The most promising targets include BMP and myostatin [105]. Indeed, medication with human recombinant BMP-2/7 and antimyostatin can help to reduce sarcopenic symptoms [106]. Cachexia is addressed with anamorelin, a ghrelin agonist, and selective androgen receptor modulator as well as anticytokines/myokines [107]. Another factor involved in muscle healing seems to be TGF-*β*. Increased TGF-*β*1 levels, which could be detected after the use of nonsteroidal anti-inflammatory drugs, helped to regenerate muscle tissue [108–110].

Spinal muscular atrophy arises from mutations in the survival motor neuron 1 (SMN1) gene, which often leads to the deficiency of the ubiquitous SMN protein [111]. Therefore, one of the most promising strategies is to increase the levels of full-length SMN [112]. Nusinersen is an antisense oligonucleotide drug developed for the treatment of spinal muscular atrophy (SMA), which has been approved by the US Food and Drug Administration (FDA) and European Medicines Agency (EMA) [113]. It can modulate the pre-mRNA splicing of the survival motor neuron 2 gene and showed significant improvement of muscle function after treatment. Clinical trials on infants showed significant mean improvements in developmental motor milestones including sitting, walking, and motor function [114].

### 3.4. Other Developing Techniques

The effect of heat stress on skeletal muscle regeneration was investigated in experimental rats [115]. Results showed that applying heat packs immediately after crush injury accelerated the degeneration process at the injured site, facilitated migration of macrophages, proliferation, and differentiation of satellite cells, and promoted muscle tissue regeneration.

Low-level laser therapy (LLLT) has also been evaluated as a therapeutic approach for stimulating muscle repair and recovery after endurance exercise training in rats [116]. Other results from the rat model suggest that it could also be an option to reduce fibrosis and myonecrosis triggered by bupivacaine and accelerate the muscle regeneration process [117]. As possible mechanisms, decreased inflammation and muscle creatine kinase levels are discussed. The combination of LLLT with platelet rich plasma (PRP) produced better results for promoting muscle regeneration after injuries compared to the isolated use of LLLT or PRP [118].

The effect of neuromuscular electrical stimulation (NMES) on skeletal muscle regeneration was assessed in healthy subjects. It increased the proliferation of myogenic precursor cells (MPCs) and their fusion with mature myofibers, which improved the regenerative capacity of skeletal muscle [119]. The effect on models with muscle injury or VML needs to be further investigated.

## 4. Challenges and Future Perspectives

### 4.1. Mechanical Properties of Biomaterials

Biomaterials for muscle tissue engineering and regeneration should persist long enough to support organized functional muscle regeneration and could be degraded gradually along with new tissue formation. The scaffolds created with natural polymers are usually associated with poor mechanical stiffness and rapid degradability, when not chemically crosslinked [120]. Synthetic polymers provide an artificial alternative with flexible mechanical properties [121, 122]. However, the use of synthetic scaffolds can be associated with side effects such as inhibition of cell migration and cell-to-cell communication [123].

A challenge for the near future will be to join the advantageous properties of natural and artificial polymers. Design of scaffolds combining favorable cell interaction with mechanical strength will facilitate implantation, give direct support to the tissue, and allow remodeling and therefore regeneration of the impaired tissue. Ideally these materials can then be used in combination with 3D-printing technology to tailor the scaffold based on the individual loss of muscle.

The mechanical and surface properties of the scaffold can be further engineered to affect the cell behavior in terms of adhesion, proliferation, migration, and differentiation [124]. If stem cells are seeded onto such scaffolds, they may therefore be guided to differentiate into different types of cells based on the scaffold properties [125, 126]. Moreover, degradation products from an ECM scaffold might contribute to the recruitment of host cells for tissue remodeling by chemoattraction [127]. Thus, better understanding of cell-scaffold interaction and development of a carrier scaffold that stimulates the niche environment for ongoing remodeling processes are further goals for future development in this area.

### 4.2. Vascularization in the Process of Regeneration

For engineering muscle constructs in vitro, one of the major limitations is the lack of vascularization [128]. It has been shown that myoblasts need to be within 150 *μ*m of the supply route for oxygen and nutrients (typically vessels) to survive, proliferate, and differentiate [129]. This limits the size of constructs without a functional vascular network. Insufficient vascularization can lead to nutrient deficiencies and hypoxia deeper in the scaffolds, which results in nonuniform cell differentiation and integration, and thus decreases tissue functionality [130].

Also for in vivo muscle tissue regeneration facilitated by bioengineered muscle tissue constructs, the absence of immediate blood supply is one main reason for failure [131]. Complete revascularization of scaffolds by ingrowth of bed vessels into the graft can take up to 3 weeks, which significantly limits the capacity to obtain scar free tissue regeneration [132]. An inability of fast vascularization inevitably results in cell death and in the worst case loss of the tissue [133].

In order to solve this problem, different approaches for improved vascularization are conceivable: One way is administration of growth factors like bFGF, which can accelerate neoangiogenesis in the early stages of healing [134]. Another possibility is a coculture with endothelial cells [135]. In addition, integration of vascular networks into the bioengineered scaffold by microfluidic methods or bioprinting is expected to provide solutions in the near future [128, 136–138]. Maybe the combination of several approaches will eventually solve the current vascularization deficit of the designed tissues.

### 4.3. Innervation of Regenerated Muscles

A critical step for regenerating functional muscle tissue after VML injuries is achieving de novo innervation of regenerated myofibers (e.g., reestablishment of neuromuscular junctions, NMJs); otherwise, the regenerated muscle will become atrophic [139]. In all cases of autologous muscle transplantation, the force developed following direct or nerve stimulation is weaker than normal [140]. This is partially due to increased connective tissue and the failure of regeneration of some muscles. Another critical factor is the poor reinnervation at the sites of the original NMJs, which influences the force output [24]. It is unclear to what extent the innervation of the regenerated muscles can be restored. To rebuild the NHJs in newly regenerated muscle fibers, nerves need to be regenerated and new motor endplates have to be formed. The motor endplates not only confer functional control over the newly regenerated muscles, but also influence muscle fiber type, alignment, and size [141]. So far studies on the reinnervation of skeletal muscles have been limited to in vitro coculture of muscle cells and neurons [142, 143]. Those results showed better contractile force in nerve-muscle constructs and then in muscle-only constructs. However, full reestablishment of new nerves and motor endplates within new muscles has proven difficult, which needs to be further investigated.

### 4.4. Immune System Problems with Scaffolds and Cells

Matrix derived from both allografts and xenografts is often rejected because of host immune responses arising from antigens present in the donor tissue (e.g., Gal epitope, DNA, and damage associated molecular pattern molecules) [127, 144, 145]. They are typically processed by decellularization and/or chemical crosslinking to remove or cover antigenic molecules [146]. Specific decellularization techniques seem to alleviate some of these problems for ECM [147, 148]. However, remnant DNA within biological scaffolds after decellularization can still induce inflammatory reactions following implantation [149]. The host immune response to biological scaffolds differs among the sources of the raw materials from which the ECM is harvested, the processing steps, to the intended clinical application [127]. The cellular response to porcine SIS crosslinked with carbodiimide was shown to be predominated by a neutrophilic-type response, whereas foreign-body response associated with multinucleate giant cells was observed at the surgical site implanted with human dermis and porcine dermis. The host tissue response to porcine SIS showed organized connective tissue formation and muscle cells proliferation whereas the tissue response to human dermis was predominated by a persistent low-grade chronic inflammation with fibrous connective tissue formation, which might form an adverse environment for muscle tissue regeneration [150]. Therefore, the host immune reaction to biomaterials is a challenge that needs to be overcome by either designing materials that do not elicit such effects or modulating the adverse immune response.

Also for polymeric biomaterials, immunological compatibility remains a problem and limited biocompatibility sometimes causes local morbidity and chronic inflammation [108]. One reason could be that polymeric biomaterials attract multinucleated giant cells for disintegration [151].

Whether immune activation results in tissue regeneration or scarring is determined also by the availability of a stem or progenitor cell pool [152]. The cell source seems to be important with less immunogenicity in embryonic and adult stem cells [153]. Consequently, cells isolated from cord blood and autologous stem cells would be preferred for clinical application in such materials. Induced pluripotent stem cells (iPSCs) have a wide possible range of application as their production is relatively straight forward and they can differentiate in nearly every cell type. They might be able to overcome immunogenicity and ethical concerns. However, safety concerns for the use of iPSCs in patients currently result in very high regulatory barriers that will inhibit clinical translation for the foreseeable future [154]. The interactions between immune cells and resident cells are important in skeletal muscle regeneration. Macrophages, eosinophils, and regulatory T cells have been shown to activate satellite cells, which contribute to myofibers formation after injury [155–157]. In depth understanding of the immune reactions to both biological scaffolds and transplanted cells may provide clues to therapeutic avenues to promote muscle tissue regeneration. Study of the immunomodulation by scaffolds, materials, and cells in combination with subtle signaling might provide new strategies for enhancing muscle tissue regeneration through guided cell response.

## 5. Conclusion

Skeletal muscle injury or loss occurs in many clinical situations. Surgical techniques are highly developed and can provide good results for reconstructing muscle function, if all goes well. Surgery is always associated with considerable risks and high costs and even if successful, usually better function at one location is traded for impaired function at another location that is less important for the patient. Research into tissue engineering and regenerative cell therapy may overcome these problems. Tissue engineering solutions will have to combine biomimetic scaffolds which guide muscle tissue growth with growth factors, embedded supply routes, and relevant cells. These cells will have to directly improve local myogenic cell amount in injured or atrophic muscles, which can be expected to promote muscle regeneration. Such creative solutions will have to rely on a deep understanding of the regeneration process required for functional muscle regeneration (cell response to scaffolds, vascularization, myogenesis, and innervation), which will require further studies.

## References

[1] Kwee B. J., Mooney D. J. (2017). Biomaterials for skeletal muscle tissue engineering. *Current Opinion in Biotechnology*.

[2] Tedesco F. S., Dellavalle A., Diaz-Manera J., Messina G., Cossu G. (2010). Repairing skeletal muscle: regenerative potential of skeletal muscle stem cells. *The Journal of Clinical Investigation*.

[3] Wu X., Corona B. T., Chen X., Walters T. J. (2012). A Standardized Rat Model of Volumetric Muscle Loss Injury for the Development of Tissue Engineering Therapies. *BioResearch Open Access*.

[4] Grogan B. F., Hsu J. R. (2011). Volumetric muscle loss. *American Academy of Orthopaedic Surgeon*.

[5] Corona B. T., Rivera J. C., Owens J. G., Wenke J. C., Rathbone C. R. (2015). Volumetric muscle loss leads to permanent disability following extremity trauma. *Journal of Rehabilitation Research and Development *.

[6] Pollot B. E., Corona B. T. (2016). Volumetric muscle loss. *Methods in Molecular Biology*.

[7] Quintero A. J., Wright V. J., Fu F. H., Huard J. (2009). Stem cells for the treatment of skeletal muscle injury. *Clinics in Sports Medicine*.

[8] Counsel P., Breidahl W. (2010). Muscle injuries of the lower leg. *Seminars in Musculoskeletal Radiology*.

[9] Turner N. J., Badylak S. F. (2012). Regeneration of skeletal muscle. *Cell and Tissue Research*.

[10] Saure C., Caminiti C., Weglinski J., de Castro Perez F., Monges S. (2017). Energy expenditure, body composition, and prevalence of metabolic disorders in patients with Duchenne muscular dystrophy. *Diabetes & Metabolic Syndrome: Clinical Research & Reviews*.

[11] Pansarasa O., Rossi D., Berardinelli A., Cereda C. (2014). Amyotrophic lateral sclerosis and skeletal muscle: An update. *Molecular Neurobiology*.

[12] Jani-Acsadi A., Ounpuu S., Pierz K., Acsadi G. (2015). Pediatric Charcot-Marie-Tooth Disease. *Pediatric Clinics of North America*.

[13] Yiu E. M., Kornberg A. J. (2015). Duchenne muscular dystrophy. *Journal of Paediatrics and Child Health*.

[14] Kalyani R. R., Corriere M., Ferrucci L. (2014). Age-related and disease-related muscle loss: the effect of diabetes, obesity, and other diseases. *The Lancet Diabetes & Endocrinology*.

[15] Tuffaha et al. S. H. (2016). Growth hormone therapy accelerates axonal regeneration, promotes motor reinnervation, and reduces muscle atrophy following peripheral nerve injury. *Plast Reconstr Surg*.

[16] Mase V. J., Hsu J. R., Wolf S. E. (2010). Clinical application of an acellular biologic scaffold for surgical repair of a large, traumatic quadriceps femoris muscle defect. *Orthopedics*.

[17] Merritt E. K., Cannon M. V., Hammers D. W. (2010). Repair of traumatic skeletal muscle injury with bone-marrow-derived mesenchymal stem cells seeded on extracellular matrix. *Tissue Engineering Part A*.

[18] Pannérec A., Marazzi G., Sassoon D. (2012). Stem cells in the hood: The skeletal muscle niche. *Trends in Molecular Medicine*.

[19] Trappmann B., Gautrot J. E., Connelly J. T. (2012). Extracellular-matrix tethering regulates stem-cell fate. *Nature Materials*.

[20] Gordon B. S., Kelleher A. R., Kimball S. R. (2013). Regulation of muscle protein synthesis and the effects of catabolic states. *The International Journal of Biochemistry & Cell Biology*.

[21] Chargé S. B. P., Rudnicki M. A. (2004). Cellular and molecular regulation of muscle regeneration. *Physiological Reviews*.

[22] Ten Broek R. W., Grefte S., Von Den Hoff J. W. (2010). Regulatory factors and cell populations involved in skeletal muscle regeneration. *Journal of Cellular Physiology*.

[23] Schiaffino S., Dyar K. A., Ciciliot S., Blaauw B., Sandri M. (2013). Mechanisms regulating skeletal muscle growth and atrophy. *FEBS Journal*.

[24] Wu P., Chawla A., Spinner R. J. (2014). Key changes in denervated muscles and their impact on regeneration and reinnervation. *Neural Regeneration Research*.

[25] Serrano A. L., Muñoz-Cánoves P. (2010). Regulation and dysregulation of fibrosis in skeletal muscle. *Experimental Cell Research*.

[26] Grzelkowska-Kowalczyk K., Travascio F. (2016). The importance of extracellular matrix in skeletal muscle development and function. *Composition and Function of the Extracellular Matrix in the Human Body*.

[27] Kharraz Y., Guerra J., Pessina P., Serrano A. L., Muñoz-Cánoves P. (2014). Understanding the process of fibrosis in duchenne muscular dystrophy. *BioMed Research International*.

[28] Klingler et al. W. (2012). The role of fibrosis in Duchenne muscular dystrophy. *Acta Myol*.

[29] Grasman J. M., Zayas M. J., Page R. L., Pins G. D. (2015). Biomimetic scaffolds for regeneration of volumetric muscle loss in skeletal muscle injuries. *Acta Biomaterialia*.

[30] Taudorf E. H., Danielsen P. L., Paulsen I. F. (2015). Non-ablative fractional laser provides long-term improvement of mature burn scars - A randomized controlled trial with histological assessment. *Lasers in Surgery and Medicine*.

[31] Krakowski A. C., Goldenberg A., Eichenfield L. F., Murray J.-P., Shumaker P. R. (2014). Ablative fractional laser resurfacing helps treat restrictive pediatric scar contractures. *Pediatrics*.

[32] Byrne M., O'Donnell M., Fitzgerald L., Shelley O. P. (2016). Early experience with fat grafting as an adjunct for secondary burn reconstruction in the hand: Technique, hand function assessment and aesthetic outcomes. *Burns*.

[33] Klinkenberg M., Fischer S., Kremer T., Hernekamp F., Lehnhardt M., Daigeler A. (2013). Comparison of anterolateral thigh, lateral arm, and parascapular free flaps with regard to donor-site morbidity and aesthetic and functional outcomes. *Plastic and Reconstructive Surgery*.

[34] Stevanovic M. V., Cuéllar V. G., Ghiassi A., Sharpe F. (2016). Single-stage Reconstruction of Elbow Flexion Associated with Massive Soft-Tissue Defect Using the Latissimus Dorsi Muscle Bipolar Rotational Transfer. *Plastic and Reconstructive Surgery - Global Open*.

[35] Makarewich C. A., Hutchinson D. T. (2016). Tendon Transfers for Combined Peripheral Nerve Injuries. *Hand Clinics*.

[36] Eckardt A., Fokas K. (2003). Microsurgical reconstruction in the head and neck region: An 18-year experience with 500 consecutive cases. *Journal of Cranio-Maxillo-Facial Surgery*.

[37] Estrella E. P., Montales T. D. (2016). Functioning free muscle transfer for the restoration of elbow flexion in brachial plexus injury patients. *Injury*.

[38] Bertelli J. A., Ghizoni M. F. (2016). Nerve and Free Gracilis Muscle Transfers for Thumb and Finger Extension Reconstruction in Long-standing Tetraplegia. *Journal of Hand Surgery*.

[39] Barrera-Ochoa S., Collado-Delfa J. M., Sallent A., Lluch A., Velez R. (2017). Free Neurovascular Latissimus Dorsi Muscle Transplantation for Reconstruction of Hip Abductors. *Plastic and Reconstructive Surgery - Global Open*.

[40] Maldonado A. A., Kircher M. F., Spinner R. J., Bishop A. T., Shin A. Y. (2017). Free Functioning Gracilis Muscle Transfer With and Without Simultaneous Intercostal Nerve Transfer to Musculocutaneous Nerve for Restoration of Elbow Flexion After Traumatic Adult Brachial Pan-Plexus Injury. *Journal of Hand Surgery*.

[41] Rozen S. M. (2017). Facial reanimation: basic surgical tools and creation of an effective toolbox for treating patients with facial paralysis. part a: functional muscle transfers in the long-term facial palsy patient. *Plastic and Reconstructive Surgery*.

[42] Jones C. S., Nowers J., Smart N. J., Coelho J., Watts A., Daniels I. R. (2017). Pelvic floor reconstruction with bilateral gracilis flaps following extralevator abdominoperineal excision - a video vignette. *Colorectal Disease*.

[43] Lin C., Lin Y., Chen C., Wei F. (2007). Free Functioning Muscle Transfer for Lower Extremity Post-Traumatic Composite Structure and Functional Defect. *Journal of Reconstructive Microsurgery*.

[44] Bianchi B., Copelli C., Ferrari S., Ferri A., Sesenna E. (2009). Free flaps: Outcomes and complications in head and neck reconstructions. *Journal of Cranio-Maxillo-Facial Surgery*.

[45] Wang X. H., Du J., Klein J. D., Bailey J. L., Mitch W. E. (2009). Exercise ameliorates chronic kidney disease-induced defects in muscle protein metabolism and progenitor cell function. *Kidney International*.

[46] Gregory T. M., Heckmann R. A., Francis R. S. (1995). The effect of exercise on the presence of leukocytes, erythrocytes and collagen fibers in skeletal muscle after contusion. *J Manipulative Physiol Ther*.

[47] Brutsaert et al. T. D. (2002). Regional differences in expression of VEGF mRNA in rat gastrocnemius following 1 hr exercise or electrical stimulation. *BMC Physiol*.

[48] Faria et al. F. E. (2008). The onset and duration of mobilization affect the regeneration in the rat muscle. *Histol Histopathol*.

[49] Aurora A., Garg K., Corona B. T., Walters T. J. (2014). Physical rehabilitation improves muscle function following volumetric muscle loss injury. *BMC Sports Science, Medicine and Rehabilitation*.

[50] Andrzejewski W., Kassolik K., Kobierzycki C. (2015). Increased skeletal muscle expression of VEGF induced by massage and exercise. *Folia Histochemica et Cytobiologica*.

[51] Chen Y., Sood S., Biada J., Roth R., Rabkin R. (2008). Increased workload fully activates the blunted IRS-1/PI3-kinase/Akt signaling pathway in atrophied uremic muscle. *Kidney International*.

[52] Majchrzak K. M., Pupim L. B., Flakoll P. J., Ikizler T. A. (2008). Resistance exercise augments the acute anabolic effects of intradialytic oral nutritional supplementation. *Nephrology Dialysis Transplantation *.

[53] Kopple J. D., Wang H., Casaburi R. (2007). Exercise in maintenance hemodialysis patients induces transcriptional changes in genes favoring anabolic muscle. *Journal of the American Society of Nephrology*.

[54] Zhao K. (2013). Acupuncture for the treatment of insomnia. *International Review of Neurobiology*.

[55] Haddad N. E., Palesh O. (2014). Acupuncture in the treatment of cancer-related psychological symptoms. *Integrative Cancer Therapies*.

[56] Urruela M. A., Suarez-Almazor M. E. (2012). Acupuncture in the treatment of rheumatic diseases. *Current Rheumatology Reports*.

[57] Takaoka Y., Ohta M., Ito A. (2007). Electroacupuncture suppresses myostatin gene expression: Cell proliferative reaction in mouse skeletal muscle. *Physiological Genomics*.

[58] Su Z., Robinson A., Hu L. (2015). Acupuncture plus low-frequency electrical stimulation (Acu-LFES) attenuates diabetic myopathy by enhancing muscle regeneration. *PLoS ONE*.

[59] Hu L., Klein J. D., Hassounah F. (2015). Low-frequency electrical stimulation attenuates muscle atrophy in CKD-a potential treatment strategy. *Journal of the American Society of Nephrology*.

[60] Sicari B. M., Rubin J. P., Dearth C. L. (2014). An acellular biologic scaffold promotes skeletal muscle formation in mice and humans with volumetric muscle loss. *Science Translational Medicine*.

[61] Valentin J. E., Turner N. J., Gilbert T. W., Badylak S. F. (2010). Functional skeletal muscle formation with a biologic scaffold. *Biomaterials*.

[62] Wang L., Cao L., Shansky J., Wang Z., Mooney D., Vandenburgh H. (2014). Minimally invasive approach to the repair of injured skeletal muscle with a shape-memory scaffold. *Molecular Therapy*.

[63] Cezar C. A., Mooney D. J. (2015). Biomaterial-based delivery for skeletal muscle repair. *Advanced Drug Delivery Reviews*.

[64] Hill E., Boontheekul T., Mooney D. J. (2006). Regulating activation of transplanted cells controls tissue regeneration. *Proceedings of the National Acadamy of Sciences of the United States of America*.

[65] Moon D. G., Christ G., Stitzel J. D., Atala A., Yoo J. J. (2008). Cyclic mechanical preconditioning improves engineered muscle contraction. *Tissue Engineering - Part A.*.

[66] Lesman A., Koffler J., Atlas R., Blinder Y. J., Kam Z., Levenberg S. (2011). Engineering vessel-like networks within multicellular fibrin-based constructs. *Biomaterials*.

[67] Bidarra S. J., Barrias C. C., Granja P. L. (2014). Injectable alginate hydrogels for cell delivery in tissue engineering. *Acta Biomaterialia*.

[68] Walters B. D., Stegemann J. P. (2014). Strategies for directing the structure and function of three-dimensional collagen biomaterials across length scales. *Acta Biomaterialia*.

[69] Brown A. C., Barker T. H. (2014). Fibrin-based biomaterials: modulation of macroscopic properties through rational design at the molecular level. *Acta Biomaterialia*.

[70] Boontheekul T., Hill E. E., Kong H.-J., Mooney D. J. (2007). Regulating myoblast phenotype through controlled gel stiffness and degradation. *Tissue Engineering Part A*.

[71] Kroehne V., Heschel I., Schügner F., Lasrich D., Bartsch J. W., Jockusch H. (2008). Use of a novel collagen matrix with oriented pore structure for muscle cell differentiation in cell culture and in grafts. *Journal of Cellular and Molecular Medicine*.

[72] Frey S. P., Jansen H., Raschke M. J., Meffert R. H., Ochman S. (2012). VEGF improves skeletal muscle regeneration after acute trauma and reconstruction of the limb in a rabbit model. *Clinical Orthopaedics and Related Research*.

[73] Ju Y. M., Atala A., Yoo J. J., Lee S. J. (2014). In situ regeneration of skeletal muscle tissue through host cell recruitment. *Acta Biomaterialia*.

[74] Beier J. P., Kneser U., Stern-Sträter J., Stark G. B., Bach A. D. (2004). Y Chromosome Detection of Three-Dimensional Tissue-Engineered Skeletal Muscle Constructs in a Syngeneic Rat Animal Model. *Cell Transplantation*.

[75] Grasman J. M., Do D. M., Page R. L., Pins G. D. (2015). Rapid release of growth factors regenerates force output in volumetric muscle loss injuries. *Biomaterials*.

[76] Cronin E. M., Thurmond F. A., Bassel-Duby R. (2004). Protein-coated poly(L-lactic acid) fibers provide a substrate for differentiation of human skeletal muscle cells. *Journal of Biomedical Materials Research Part B: Applied Biomaterials*.

[77] Hoque M. E., San W. Y., Wei F. (2009). Processing of polycaprolactone and polycaprolactone-based copolymers into 3D scaffolds, and their cellular responses. *Tissue Engineering Part: A*.

[78] Guex A. G., Birrer D. L., Fortunato G., Tevaearai H. T., Giraud M.-N. (2013). Anisotropically oriented electrospun matrices with an imprinted periodic micropattern: A new scaffold for engineered muscle constructs. *Biomedical Materials*.

[79] Yang J., Jao B., Mcnally A. K., Anderson J. M. (2014). In vivo quantitative and qualitative assessment of foreign body giant cell formation on biomaterials in mice deficient in natural killer lymphocyte subsets, mast cells, or the interleukin-4 receptor*α* and in severe combined immunodeficient mice. *Journal of Biomedical Materials Research Part A*.

[80] Turner N. J., Yates A. J., Weber D. J. (2010). Xenogeneic extracellular matrix as an inductive scaffold for regeneration of a functioning musculotendinous junction. *Tissue Engineering Part A*.

[81] Merritt E. K., Hammers D. W., Tierney M., Suggs L. J., Walters T. J., Farrar R. P. (2010). Functional assessment of skeletal muscle regeneration utilizing homologous extracellular matrix as scaffolding. *Tissue Engineering Part A*.

[82] DeQuach J. A., Lin J. E., Cam C. (2012). Injectable skeletal muscle matrix hydrogel promotes neovascularization and muscle cell infiltration in a hindlimb ischemia model. *European Cells and Materials*.

[83] Garg K., Ward C. L., Rathbone C. R., Corona B. T. (2014). Transplantation of devitalized muscle scaffolds is insufficient for appreciable de novo muscle fiber regeneration after volumetric muscle loss injury. *Cell and Tissue Research*.

[84] Vannozzi L., Ricotti L., Santaniello T. (2017). 3D porous polyurethanes featured by different mechanical properties: Characterization and interaction with skeletal muscle cells. *Journal of the Mechanical Behavior of Biomedical Materials*.

[85] Brown B. N., Valentin J. E., Stewart-Akers A. M., McCabe G. P., Badylak S. F. (2009). Macrophage phenotype and remodeling outcomes in response to biologic scaffolds with and without a cellular component. *Biomaterials*.

[86] Collins C. A., Olsen I., Zammit P. S. (2005). Stem cell function, self-renewal, and behavioral heterogeneity of cells from the adult muscle satellite cell niche. *Cell*.

[87] MacHingal M. A., Corona B. T., Walters T. J. (2011). A tissue-engineered muscle repair construct for functional restoration of an irrecoverable muscle injury in a murine model. *Tissue Engineering Part A*.

[88] Lee J.-H., Kosinski P. A., Kemp D. M. (2005). Contribution of human bone marrow stem cells to individual skeletal myotubes followed by myogenic gene activation. *Experimental Cell Research*.

[89] Sacco A., Mourkioti F., Tran R. (2010). Short telomeres and stem cell exhaustion model duchenne muscular dystrophy in mdx/mTR mice. *Cell*.

[90] Cerletti M., Jurga S., Witczak C. A. (2008). Highly efficient, functional engraftment of skeletal muscle stem cells in dystrophic muscles. *Cell*.

[91] Montarras D., Morgan J., Colins C. (2005). Developmental biology: direct isolation of satellite cells for skeletal muscle regeneration. *Science*.

[92] Meng J., Muntoni F., Morgan J. E. (2011). Stem cells to treat muscular dystrophies - Where are we?. *Neuromuscular Disorders*.

[93] Borselli C., Cezar C. A., Shvartsman D., Vandenburgh H. H., Mooney D. J. (2011). The role of multifunctional delivery scaffold in the ability of cultured myoblasts to promote muscle regeneration. *Biomaterials*.

[94] Wolf M. T., Daly K. A., Reing J. E., Badylak S. F. (2012). Biologic scaffold composed of skeletal muscle extracellular matrix. *Biomaterials*.

[95] Miller R., Sharma K., Pavlath G. Myoblast implantation in Duchenne muscular dystrophy: The San Francisco study. *Muscle & Nerve*.

[96] Sampaolesi M., Blot S., D'Antona G. (2006). Mesoangioblast stem cells ameliorate muscle function in dystrophic dogs. *Nature*.

[97] Fuoco C., Salvatori M., Biondo A. (2012). Injectable polyethylene glycol-fibrinogen hydrogel adjuvant improves survival and differentiation of transplanted mesoangioblasts in acute and chronic skeletal-muscle degeneration. *Skeletal Muscle*.

[98] Zhang et al. C. (2005). Therapy of Duchenne muscular dystrophy with umbilical cord blood stem cell transplantation. *Zhonghua Yi Xue Yi Chuan Xue Za Zhi*.

[99] Hammers D. W., Sarathy A., Pham C. B., Drinnan C. T., Farrar R. P., Suggs L. J. (2012). Controlled release of IGF-I from a biodegradable matrix improves functional recovery of skeletal muscle from ischemia/reperfusion. *Biotechnology and Bioengineering*.

[100] Borselli C., Storrie H., Benesch-Lee F. (2010). Functional muscle regeneration with combined delivery of angiogenesis and myogenesis factors. *Proceedings of the National Acadamy of Sciences of the United States of America*.

[101] Shvartsman D., Storrie-White H., Lee K. (2014). Sustained delivery of VEGF maintains innervation and promotes reperfusion in ischemic skeletal muscles via NGF/GDNF signaling. *Molecular Therapy*.

[102] Rybalko V. Y., Pham C. B., Hsieh P.-L. (2015). Controlled delivery of SDF-1*α* and IGF-1: CXCR4+ cell recruitment and functional skeletal muscle recovery. *Biomaterials Science*.

[103] Hwang J. H., Kim I. G., Piao S. (2013). Combination therapy of human adipose-derived stem cells and basic fibroblast growth factor hydrogel in muscle regeneration. *Biomaterials*.

[104] Ho T.-C., Chiang Y.-P., Chuang C.-K. (2015). PEDF-derived peptide promotes skeletal muscle regeneration through its mitogenic effect on muscle progenitor cells. *American Journal of Physiology-Cell Physiology*.

[105] Saul D S. A., Kosinsky R. L. (2017). Why age matters: inflammation, cancer and hormones in the development of sarcopenia. *Journal of Osteoporosis and Physical Activity*.

[106] Scimeca M., Piccirilli E., Mastrangeli F. (2017). Bone Morphogenetic Proteins and myostatin pathways: Key mediator of human sarcopenia. *Journal of Translational Medicine*.

[107] Molfino A., Amabile M. I., Rossi Fanelli F., Muscaritoli M. (2016). Novel therapeutic options for cachexia and sarcopenia. *Expert Opinion on Biological Therapy*.

[108] Berebichez-Fridman R., Gómez-García R., Granados-Montiel J. (2017). The Holy Grail of Orthopedic Surgery: Mesenchymal Stem Cells - Their Current Uses and Potential Applications. *Stem Cells International*.

[109] Tseng S. S., Lee M. A., Reddi A. H. (2008). Nonunions and the potential of stem cells in fracture-healing. *The Journal of Bone & Joint Surgery—American Volume*.

[110] Qu-Petersen Z., Deasy B., Jankowski R. (2002). Identification of a novel population of muscle stem cells in mice: potential for muscle regeneration. *The Journal of Cell Biology*.

[111] Monani U. R. (2005). Spinal muscular atrophy: A deficiency in a ubiquitous protein; a motor neuron-specific disease. *Neuron*.

[112] Parente V., Corti S. (2018). Advances in spinal muscular atrophy therapeutics. *Therapeutic Advances in Neurological Disorders*.

[113] Mercuri E., Darras B. T., Chiriboga C. A. (2018). Nusinersen versus Sham Control in Later-Onset Spinal Muscular Atrophy. *The New England Journal of Medicine*.

[114] Finkel R. S., Chiriboga C. A., Vajsar J. (2016). Treatment of infantile-onset spinal muscular atrophy with nusinersen: a phase 2, open-label, dose-escalation study. *The Lancet*.

[115] Takeuchi K., Hatade T., Wakamiya S., Fujita N., Arakawa T., Miki A. (2014). Heat stress promotes skeletal muscle regeneration after crush injury in rats. *Acta Histochemica*.

[116] Assis L., Yamashita F., Magri A. M. P., Fernandes K. R., Yamauchi L., Renno A. C. M. (2015). Effect of low-level laser therapy (808 nm) on skeletal muscle after endurance exercise training in rats. *Brazilian Journal of Physical Therapy*.

[117] Alessi Pissulin C. N., Henrique Fernandes A. A., Sanchez Orellana A. M., Rossi e Silva R. C., Michelin Matheus S. M. (2017). Low-level laser therapy (LLLT) accelerates the sternomastoid muscle regeneration process after myonecrosis due to bupivacaine. *Journal of Photochemistry and Photobiology B: Biology*.

[118] Garcia T. A., Camargo R. C., Koike T. E., Ozaki G. A., Castoldi R. C., Camargo Filho J. C. (2017). Histological analysis of the association of low level laser therapy and platelet-rich plasma in regeneration of muscle injury in rats. *Brazilian Journal of Physical Therapy*.

[119] Filippo E. S. D., Mancinelli R., Marrone M. (2017). Neuromuscular electrical stimulation improves skeletal muscle regeneration through satellite cell fusion with myofibers in healthy elderly subjects. *Journal of Applied Physiology*.

[120] Chaudhari A. A., Vig K., Baganizi D. R. (2016). Future prospects for scaffolding methods and biomaterials in skin tissue engineering: a review. *International Journal of Molecular Sciences*.

[121] Liu J., Zheng H., Poh P., Machens H., Schilling A. (2015). Hydrogels for Engineering of Perfusable Vascular Networks. *International Journal of Molecular Sciences*.

[122] Guo B., Ma P. X. (2014). Synthetic biodegradable functional polymers for tissue engineering: a brief review. *SCIENCE CHINA Chemistry*.

[123] Reing J. E., Zhang L., Myers-Irvin J. (2009). Degradation products of extracellular matrix affect cell migration and proliferation. *Tissue Engineering Part A*.

[124] Griffin M., Nayyer L., Butler P. E., Palgrave R. G., Seifalian A. M., Kalaskar D. M. (2016). Development of mechano-responsive polymeric scaffolds using functionalized silica nano-fillers for the control of cellular functions. *Nanomedicine: Nanotechnology, Biology and Medicine*.

[125] Yim E. K. F., Sheetz M. P. (2012). Force-dependent cell signaling in stem cell differentiation. *Stem Cell Research & Therapy*.

[126] Ghassemi S., Meacci G., Liu S. (2012). Cells test substrate rigidity by local contractions on submicrometer pillars. *Proceedings of the National Acadamy of Sciences of the United States of America*.

[127] Badylak S. F., Gilbert T. W. (2008). Immune response to biologic scaffold materials. *Seminars in Immunology*.

[128] Liu J., Zheng H., Krempl F., Su L., Machens H.-G., Schilling A. F. (2016). Open source 3D-printing approach for economic and fast engineering of perfusable vessel-like channels within cell-laden hydrogels. *3D Printing and Additive Manufacturing*.

[129] Dennis R. G., Kosnik P. E. (2000). Excitability and isometric contractile properties of mammalian skeletal muscle constructs engineered in vitro. *In Vitro Cellular & Developmental Biology - Animal*.

[130] Lee C.-H., Chang S.-H., Chen W.-J. (2015). Augmentation of diabetic wound healing and enhancement of collagen content using nanofibrous glucophage-loaded collagen/PLGA scaffold membranes. *Journal of Colloid and Interface Science*.

[131] Bi H., Jin Y. (2013). Current progress of skin tissue engineering: Seed cells, bioscaffolds, and construction strategies. *Burns & Trauma*.

[132] Nyame T. T., Chiang H. A., Leavitt T., Ozambela M., Orgill D. P. (2015). Tissue-Engineered Skin Substitutes. *Plastic and Reconstructive Surgery*.

[133] Varkey M., Ding J., Tredget E. (2015). Advances in skin substitutes—potential of tissue engineered skin for facilitating anti-fibrotic healing. *Journal of Functional Biomaterials*.

[134] Thomopoulos et al. S. (2010). The effects of exogenous basic fibroblast growth factor on intrasynovial flexor tendon healing in a canine model. *J Bone Joint Surg Am*.

[135] Haro H., Kato T., Komori H., Osada M., Shinomiya K. (2002). Vascular endothelial growth factor (VEGF)-induced angiogenesis in herniated disc resorption. *Journal of Orthopaedic Research*.

[136] Nashimoto Y., Hayashi T., Kunita I. (2017). Integrating perfusable vascular networks with a three-dimensional tissue in a microfluidic device. *Integrative Biology*.

[137] Gudapati H., Dey M., Ozbolat I. (2016). A comprehensive review on droplet-based bioprinting: Past, present and future. *Biomaterials*.

[138] DiVito K. A., Daniele M. A., Roberts S. A., Ligler F. S., Adams A. A. (2017). Microfabricated blood vessels undergo neoangiogenesis. *Biomaterials*.

[139] Järvinen T. A., Järvinen T. L., Kääriäinen M. (2007). Muscle injuries: optimising recovery. *Best Practice & Research Clinical Rheumatology*.

[140] Degreef I., Debeer P., Van Herck B., Van Den Eeden E., Peers K., De Smet L. (2005). Treatment of irreparable rotator cuff tears by latissimus dorsi muscle transfer. *Acta Orthopædica Belgica*.

[141] Chen D., Chen S., Wang W. (2010). Functional modulation of satellite cells in long-term denervated human laryngeal muscle. *The Laryngoscope*.

[142] Larkin L. M., Van Der Meulen J. H., Dennis R. G., Kennedy J. B. (2006). Functional evaluation of nerve-skeletal muscle constructs engineered in vitro. *In Vitro Cellular & Developmental Biology - Animal*.

[143] Das M., Rumsey J. W., Gregory C. A. (2007). Embryonic motoneuron-skeletal muscle co-culture in a defined system. *Neuroscience*.

[144] Burd A., Chiu T. (2005). Allogenic skin in the treatment of burns. *Clinics in Dermatology*.

[145] Lotze M. T., Deisseroth A., Rubartelli A. (2007). Damage associated molecular pattern molecules. *Clinical Immunology*.

[146] Gilbert T. W., Sellaro T. L., Badylak S. F. (2006). Decellularization of tissues and organs. *Biomaterials*.

[147] Bottagisio M., Pellegata A. F., Boschetti F., Ferroni M., Moretti M., Lovati A. B. (2016). A new strategy for the decellularisation of large equine tendons as biocompatible tendon substitutes. *European Cells and Materials*.

[148] Kaully T., Kaufman-Francis K., Lesman A., Levenberg S. (2009). Vascularization—the conduit to viable engineered tissues. *Tissue Engineering Part B: Reviews*.

[149] Zheng M.-H., Chen J., Kirilak Y., Willers C., Xu J., Wood D. (2005). Porcine small intestine submucosa (SIS) is not an acellular collagenous matrix and contains porcine DNA: possible implications in human implantation. *Journal of Biomedical Materials Research Part B: Applied Biomaterials*.

[150] Badylak S., Kokini K., Tullius B., Simmons-Byrd A., Morff R. (2002). Morphologic study of small intestinal submucosa as a body wall repair device. *Journal of Surgical Research*.

[151] Al-Maawi S., Orlowska A., Sader R., James Kirkpatrick C., Ghanaati S. (2017). In vivo cellular reactions to different biomaterials—Physiological and pathological aspects and their consequences. *Seminars in Immunology*.

[152] Aurora A. B., Olson E. N. (2014). Immune modulation of stem cells and regeneration. *Cell Stem Cell*.

[153] Bifari F. (2010). Immunological properties of embryonic and adult stem cells. *World Journal of Stem Cells*.

[154] Yang et al. R. (2014). Generation of folliculogenic human epithelial stem cells from induced pluripotent stem cells. *Nat Commun*.

[155] Arnold L., Henry A., Poron F. (2007). Inflammatory monocytes recruited after skeletal muscle injury switch into antiinflammatory macrophages to support myogenesis. *The Journal of Experimental Medicine*.

[156] Heredia J. E., Mukundan L., Chen F. M. (2013). Type 2 innate signals stimulate fibro/adipogenic progenitors to facilitate muscle regeneration. *Cell*.

[157] Burzyn D., Kuswanto W., Kolodin D. (2013). A special population of regulatory T cells potentiates muscle repair. *Cell*.

